# Functional enhancement of cantaloupe rind powder by enzymatic treatment and its application in fiber-enriched cookies

**DOI:** 10.1039/d6ra05044a

**Published:** 2026-08-04

**Authors:** Thi Quynh Ngoc Nguyen, Thi Ho Thanh Dong, Le Trong Nghia Tran, Hai Duong Nguyen, Van Viet Man Le

**Affiliations:** a Faculty of Chemical Engineering, Ho Chi Minh City University of Technology (HCMUT) 268 Ly Thuong Kiet Street, Dien Hong Ward Ho Chi Minh City Vietnam lvvman@hcmut.edu.vn; b Vietnam National University-Ho Chi Minh City (VNU-HCM) Linh Xuan Ward Ho Chi Minh City Vietnam

## Abstract

Elevated postprandial blood glucose is a key contributor to metabolic disorders, including type 2 diabetes. Although dietary fiber is known to modulate glucose absorption and improve glycemic response, its intake remains below recommended levels in many populations. This has driven interest in fiber fortification of widely consumed foods such as cookies. This study investigated the effects of cellulase–xylanase treatment of cantaloupe rind powder on its composition and functional properties, and evaluated cookies formulated with 20% enzyme-treated cantaloupe rind powder (ETCP) substitution against those made with untreated cantaloupe rind powder (UTCP) and control formulations. The ETCP exhibited higher soluble dietary fiber level, increased water- and oil-holding capacities, improved glucose and cholesterol adsorption capacities, while promoting the enhanced growth of *Lactobacillus acidophilus*, along with a more porous microstructure. However, the total phenolic content and antioxidant capacity decreased. The ETCP-fortified dough showed improved viscoelasticity (higher *G*′, lower tan *δ*) and greater hardness than the UTCP counterpart. Both ETCP and UTCP increased dietary fiber, antioxidant properties, and hardness of cookies, but UTCP showed a stronger effect. Although both powders reduced overall sensory acceptability relative to the control, the ETCP resulted in a milder decline. Importantly, both formulations reduced the estimated glycemic index (eGI), with ETCP showing a greater reduction. These findings demonstrate that enzymatic modification can valorize cantaloupe rind into a functional ingredient for fiber-enriched cookies with improved glycemic response.

## Abstract 2

Elevated postprandial blood glucose is a key contributor to metabolic disorders, including type 2 diabetes.

## Graphical abstract

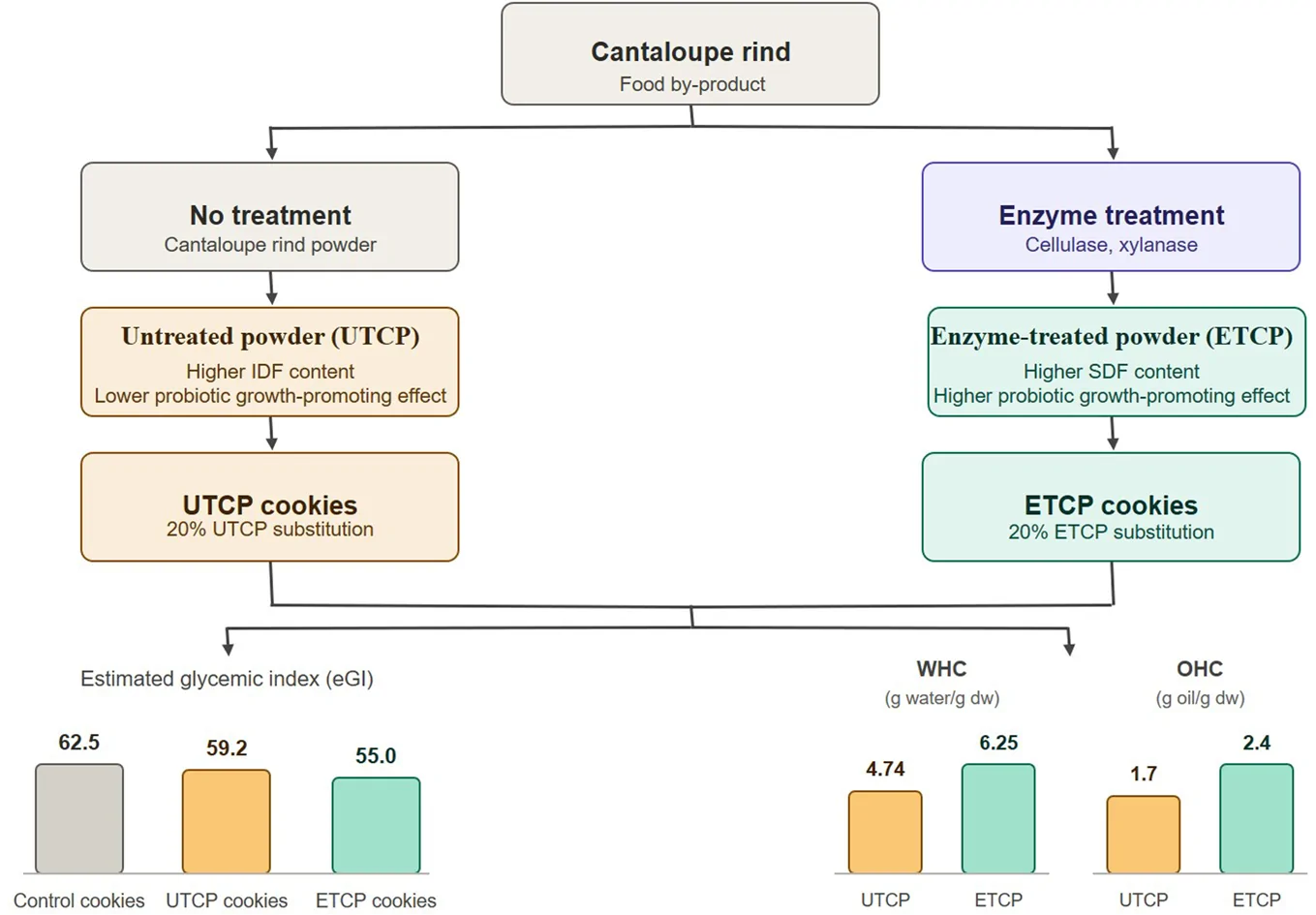

## 1. Introduction

The prevalence of metabolic disorders worldwide, particularly type 2 diabetes, has risen sharply over recent decades and represents a growing public health issue. According to the International Diabetes Federation Diabetes Atlas, an estimated 589 million adults worldwide were affected by diabetes in 2024, representing about 11.1% of the adult population, with this number projected to rise to 853 million by 2050.^1^ Elevated postprandial blood glucose is a key risk factor driving these conditions, contributing to insulin resistance, cardiovascular complications, and other metabolic disorders.^2^ Consequently, there is growing interest in dietary strategies to attenuate glycemic response and support metabolic health.

Dietary fiber (DF) is well established as a functional dietary component that contributes to the regulation of blood glucose levels. Its ability to inhibit starch hydrolysis, reduce glucose absorption, and modulate gastrointestinal transit contributes to a lower glycemic index (GI) in foods. In addition, dietary fiber plays a crucial role in maintaining a balanced and healthy gut microbiota, further supporting metabolic regulation.^3^ Despite these benefits, dietary fiber intake remains insufficient in many populations,^4^ prompting increased research interest in the fortification of commonly consumed foods. Based on water solubility, dietary fiber is commonly differentiated into soluble dietary fiber (SDF) and insoluble dietary fiber (IDF). While both types of DF contribute to health benefits, SDF generally exhibits superior functionality for glycemic control.^5,6^ The viscous nature of soluble dietary fiber (SDF) restricts enzyme–substrate interactions, which slows the digestion of complex nutrients, delays gastric emptying, reduces the rate of glucose absorption at the brush border.^7,8^ Additionally, SDF is readily metabolized by the gut microbiota, producing short-chain fatty acids (SCFAs) that regulate glucose metabolism and enhance insulin sensitivity.^9,10^ In contrast, insoluble dietary fiber (IDF) mainly enhances fecal bulk and accelerates intestinal transit, with relatively limited effects on nutrient digestion and absorption.^11^ In food matrices, fibers with a higher SDF content generally exhibit better compatibility with the matrix, often resulting in better texture and sensory properties, whereas higher levels of IDF may increase hardness or impart a gritty mouthfeel.^12–14^

To enhance the proportion of SDF in fiber-rich ingredients, several technical approaches have been explored, including physical, chemical, enzymatic and biological methods.^6^ Among these, enzymatic treatment has gained particular attention due to its specificity, mild processing conditions, and ability to selectively convert IDF into SDF.^6^ Enzymes such as cellulase and xylanase can effectively degrade structural polysaccharides, thereby enhancing SDF content and functional properties such as oil-holding capacity (OHC), water-holding capacity (WHC), glucose adsorption capacity (GAC), and cholesterol adsorption capacity (CAC).^15^

At the same time, the valorization of agricultural/food by-products has emerged as an important strategy for sustainable food production.^16^ Cantaloupe rind, a by-product of fruit processing, is often discarded despite being a rich source of dietary fiber and antioxidants.^17^ Transforming this underutilized material into a value-added functional ingredient not only reduces waste but also contributes to the development of healthier food products. Enzymatic treatment of cantaloupe rind powder with cellulase and xylanase has been reported to significantly enhance its SDF content, resulting in improved product overall quality when incorporated into probiotic yogurt compared to untreated powder.^13^

Cookies, as popular baked goods with a long shelf life, low moisture content, and broad consumer acceptance across age groups, are nonetheless typically low in dietary fiber, making fiber fortification a practical strategy for delivering fiber-rich ingredients into the diet through this widely consumed food product. Established commercial fiber ingredients such as oat fiber, citrus fiber, inulin, resistant starch, or sugar beet fiber can be used to fortify cookies and enrich their fiber content; compared with these, cantaloupe rind offers the added benefit of valorizing an agricultural by-product, while intrinsically carrying phenolic and other bioactive compounds^17^ from the fruit matrix that may confer additional benefits beyond fiber enrichment alone. While the previous study demonstrated the feasibility of incorporating enzymatically treated cantaloupe rind powder (ETCP) into a high-moisture, fermented dairy gel matrix (yogurt),^13^ its performance in a low-moisture, baked, starch- and gluten-based matrix subjected to high-temperature processing, like a cookie system, remains unexplored. This shift raises distinct technical questions not addressed previously, including how ETCP affects gluten network development and dough viscoelasticity, how its compositional changes compared to the untreated powder affect cookie textural and structural attributes, the estimated glycemic index, and the overall acceptability of a cookie product. The present study therefore provides new mechanistic and application-specific insight into the performance of ETCP in a technologically distinct cookie matrix.

Accordingly, this study aimed to evaluate the effects of enzymatic treatment using a mixture of cellulase and xylanase on the chemical composition and functional properties of cantaloupe rind powder. Furthermore, the impact of substituting 20% of wheat flour with both enzyme-treated and untreated cantaloupe rind powder in a cookie formula was evaluated, with particular emphasis on dough rheology, physical characteristics, nutritional profile, sensory acceptability, and glycemic response of the cookies. It is hypothesized that enzymatic modification will enhance the functional performance and sensory compatibility of the cantaloupe rind powder, thereby enabling its effective utilization as a novel functional ingredient for the development of fiber-enriched cookies with improved quality attributes and reduced glycemic impact.

## 2. Material and method

### 2.1 Materials

All chemicals and reagents were obtained from Merck (Darmstadt, Germany) or Sigma (St. Louis, USA) unless otherwise indicated. Ultraflo Max, containing *endo*-1,4-β-xylanase (250 U g^−1^) and *endo*-1,3(4)-β-glucanase (700 U g^−1^) was used for cantaloupe rind powder treatment; additionally, protease, amyloglucosidase, and α-amylase were used for dietary fiber and starch analyses; these enzyme preparations were all supplied by Novozymes (Bagsværd, Denmark). Pepsin and pancreatin for the *in vitro* digestion assay were obtained from HiMedia (Mumbai, India). Analytical-grade solvents, including diethyl ether and absolute ethanol, were purchased from Chemsol Vina (Vietnam). All ingredients required for cookie formulation were acquired from a local supermarket.

### 2.2 Methods

#### 2.2.1. Preparation of untreated and enzyme-treated cantaloupe rind powder (UTCP and ETCP)

##### 2.2.1.1. Preparation of UTCP

The cantaloupe rinds used in this study were obtained from cantaloupe fruits that originated from a farm in Ben Cat ward, Ho Chi Minh city. The peels were first rinsed thoroughly with tap water, after which the outer tough layer was removed. The remaining green rind was cut into 2 mm-thick slices, blanched at 90 °C for 30 s, and subsequently dried at 60 °C until the moisture content reached 10–13%. The dried material was then ground and passed through a 70-mesh (210 µm) sieve. The obtained powder was packed in plastic bags and stored at 4 °C until further use.

##### 2.2.1.2. Preparation of ETCP

The enzymatic treatment was carried out based on a previously reported method.^13^ About 10 g UTCP was dispersed in deionized water at a liquid-to-solid ratio of 15 : 1 (v : w). The suspension was then exposed to microwave irradiation in a household microwave oven at 1100 W for 2 min. The pH of the suspension, measured at 5.0–5.5, fell within the manufacturer-specified optimal working range (pH 4.0–6.5) for Ultraflo Max enzyme preparation, and was not further adjusted. After cooling to ambient temperature, Ultraflo Max was added at a dosage of 22 U cellulase and 8 U xylanase per gram of UTCP dry weight. The mixture was incubated at 55 °C for 8 h, followed by enzyme inactivation at 95 °C for 15 min.

The treated powder was recovered by introducing ethanol into the mixture at a 4 : 1 (v : v) ratio. Following phase separation by vacuum filtration, the solid fraction was recovered and subjected to drying at 60 °C to reach the target moisture content of 10–13%. The dried material was then ground and sieved through a 70-mesh screen. The final product was stored at 4 °C and designated as enzyme-treated cantaloupe rind powder (ETCP).

#### 2.2.2. Cookie preparation

The formulation of each cookie batch is presented in Table 1. To produce cookies fortified with UTCP and ETCP, 20% of the wheat flour was replaced with the corresponding cantaloupe rind powders, with the ratios of the remaining ingredients held unchanged. This substitution level was selected to balance two goals: sufficiently enhancing the dietary fiber content of the cookies to meet the minimum 6 g/100 g threshold required for a “high in fiber” claim under Codex Alimentarius guidelines, while preserving acceptable dough handling properties and cookie texture. The cookie making procedure followed a previous reported method in the study by Ngo *et al.*.^18^

**Table 1:** Ingredient formulation of control and fortified cookies^a^

Ingredients	Control cookies	Weight (g)	ETCP-fortified cookies
		UTCP-fortified cookies	
Wheat flour	75.0	60.0	60.0
UTCP	0	15.0	0
ETCP	0	0	15.0
Butter	35.0	35.0	35.0
Egg	23.3	23.3	23.3
Isomalt	23.3	23.3	23.3
Water	6.5	6.5	6.5
Baking soda	0.8	0.8	0.8
Salt	0.33	0.33	0.33
Vani	0.3	0.3	0.3
Acesulfame K	0.09	0.09	0.09

a UTCP: untreated cantaloupe rind powder; ETCP: enzyme-treated cantaloupe rind powder.

#### 2.2.3. Chemical composition

Protein and starch content was analyzed by the Kjeldahl method (AOAC 979.09) and 3,5-dinitrosalicylic acid (DNS) method^19^ (AOAC 996.11), respectively, while lipid content was determined by Soxhlet extraction using diethyl ether (AOAC 920.396). Ash content was determined by incineration at 600 °C for 18 h in a muffle furnace (Lenton Co., UK) according to AOAC 923.03. Contents of soluble dietary fiber and insoluble dietary fiber were quantified following AOAC methods 993.19 and 991.42, respectively, while total dietary fiber (TDF) was computed as their summation.

#### 2.2.4. Water holding capacity (WHC) and oil holding capacity (OHC)

WHC was determined by dispersing 1 g of sample in 10 mL of distilled water and allowing it to hydrate for 2 h. The mixture was then centrifuged (Sigma 2–16 KL, rotor 12 181, Osterode, Germany) at 3000×*g* for 20 min at room temperature. After removal of the supernatant, the remaining pellet was weighed. WHC was expressed as grams of retained water per gram of dry sample (g g^−1^ d.w). OHC was measured using the same procedure, with soybean oil used in place of water.

#### 2.2.5. Glucose adsorption capacity (GAC)

GAC of UTCP and ETCP was evaluated based on a previously reported method with minor modifications.^20^ In brief, 1.0 g of each powder was mixed with 10 mL of glucose solution (1 g L^−1^) and incubated at 37 °C for 6 h. After incubation, the suspension was centrifuged at 3000 rpm for 15 min. The residual glucose level in the supernatant was quantified by the DNS method. GAC was calculated as the reduction in glucose content in the solution before and after incubation with the sample and expressed as mg glucose/g d.w (mg g^−1^ d.w).

#### 2.2.6. Cholesterol adsorption capacity (CAC)

CAC of UTCP and ETCP was determined according to method described by Y. Zheng *et al.*^21^ with slight modifications. About 1 g of the sample was mixed with 25 mL cholesterol solution (0.5 g L^−1^) at pH 7.0 which simulated the pH conditions in the small intestine. The mixture was incubated at 37 °C in a water-bath incubator for 2 h and then centrifuged at 3000 rpm for 15 min. The cholesterol in supernatant was determined at 550 nm by using *O*-phthalaldehyde.^22^ GAC was calculated as the reduction in cholesterol content in the solution before and after incubation with the sample and expressed as mg cholesterol/g d.w (mg g^−1^).

#### 2.2.7. Growth promoting effect on *Lactobacillus acidophilus*

The growth-promoting effects of UTCP and ETCP on *Lactobacillus acidophilus* ATCC 4356 were evaluated using glucose-free MRS media supplemented with these powders. The strain was activated in MRS broth at 37 °C for 24 h and subsequently inoculated into fresh media to obtain the same initial cell density of approximately 10^5^ CFU mL^−1^. The experimental design included MRS broth with 2% (w/v) d-glucose as a positive control, glucose-free MRS as a negative control, and glucose-free MRS supplemented with UTCP or ETCP at 2%, 3%, or 4% (w/v). All cultures were incubated at 37 °C. Viable cell counts were determined after 24 and 48 h by serial dilution and pour plating on MRS agar. The pH and total titratable acidity (TA) of the cultures were measured concurrently.^23^

#### 2.2.8. Microstructural analysis by scanning electron microscopy

The microstructures of UTCP, ETCP powders, and cookie samples were observed using a S-4800 scanning electron microscope (SEM; Hitachi, Tokyo, Japan) at an accelerating voltage of 10 kV. Magnifications of 1500× and 100× were used for the powder samples and cookie samples, respectively.

#### 2.2.9. Dough rheology

Dough rheological properties were evaluated using an MCR302 rheometer (Anton Paar, Ostfildern, Germany) fitted with a parallel-plate system (25 mm diameter) at 25 °C. The viscoelastic property of the dough was evaluated by frequency sweep testing in the linear viscoelastic regime. Measurements were performed at a constant strain amplitude of 0.1% across angular frequencies from 0.1 to 10 rad s^−1^. Storage modulus (*G*′), loss modulus (*G*″), and loss tangent (tan *δ* = *G*″/*G*′) values were recorded to characterize the viscoelastic characteristics of the dough.

#### 2.2.10. Dough and cookie texture

The textural characteristics of both dough and baked cookies were determined using a TA-XT plusC texture analyzer (Stable Micro Systems, Surrey, UK). Texture profile analysis (TPA) was performed on dough using a 25 mm cylindrical probe, while cookie samples were subjected to a three-point bending test with an HDP/3 PB probe. Hardness and adhesiveness (dough) together with hardness and fracturability (cookies) were calculated from the force – time data.

#### 2.2.11. Cookie dimension

Cookie dimensions (thickness and diameter) were measured using a vernier caliper. Thickness (*T*) was evaluated by measuring the height of six stacked cookies. The diameter (*D*) of individual cookies was determined at two perpendicular positions and the mean value was used. Spread factor (SF) was subsequently calculated by dividing the average diameter by the thickness (*D*/*T*).

#### 2.2.12. Color

Color parameters were measured using a CR-300 colorimeter (Konica Minolta) and results were expressed in the CIE Lab* color space (L*, a*, and b*). The total color difference (Δ*E*) was calculated according to eqn (1), based on the differences in L*, a*, and b* values between samples.1
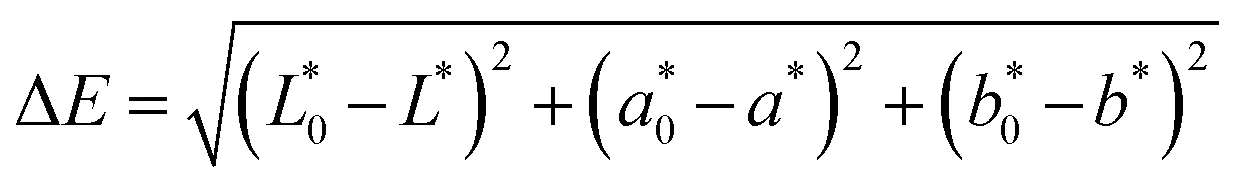
where: 
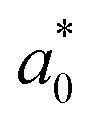
, 
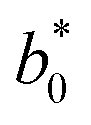
 and 
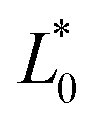
: the color values of the control cookies a*, b* and L*: the color values of the fortified cookies.

#### 2.2.13. Estimated glycemic index (eGI)

The *in vitro* glycemic index of cookie samples was evaluated based on a modified procedure reported by.^24^ Briefly, approximately 2 g of each cookie sample or white bread (reference) was homogenized with 30 mL of distilled water. The mixture pH was adjusted to 2.5 using 1 M HCl, followed by addition of 1 mL pepsin solution (10% w/v in 0.05 M HCl) to simulate gastric condition. The mixture was then shaken at 120 rpm and 37 °C for 30 min. After that, the pH was neutralized to 6.9 using 1 M NaHCO_3_ to mimic intestinal conditions. Subsequently, 5 mL of pancreatin and 0.1 mL of amyloglucosidase solution (2.5% w/v in 0.01 M phosphate buffer, pH 6.9) were added. The digestion was continued at 37 °C with constant agitation (120 rpm). Samples were collected at 0–240 min intervals, immediately stopped with ethanol (1 : 4 ratio), centrifuged (3000×*g*, 15 min), and the supernatant used for reducing sugar quantification by the DNS assay.^19^

The starch hydrolysis rate during digestion was plotted against time, and the area under the curve (AUC) was calculated. The hydrolysis index (HI) was subsequently determined according to eqn (2).2
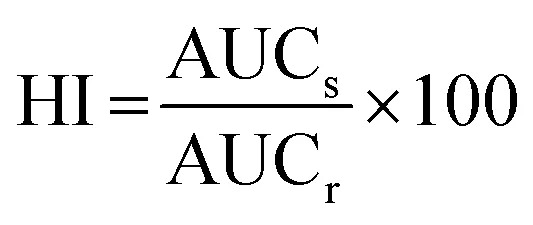


AUC_s_ and AUC_r_ correspond to the areas under the respective digestion curves of the test cookie and the white bread reference. The estimated glycemic index (eGI) was then computed from the hydrolysis index according to eqn (3) reported by Goñi *et al.*:^25^

3 eGI = 39.71 + 0.549 HI

To express the glycemic index relative to a glucose reference, the calculated values were multiplied by a conversion factor of 0.71 as described by Flavel *et al.*.^26^

#### 2.2.14. Sensory evaluation

Sensory analysis was carried out with 60 untrained participants (aged 18–25) recruited from Ho Chi Minh City University of Technology. All participants provided informed consent before the test. Cookie samples were coded with random three-digit numbers and presented individually in a randomized order. Panelists were given drinking water to rinse their palate between samples. Overall acceptability was rated on a 9-point hedonic scale, where 1 indicated “extremely dislike” and 9 indicated “extremely like”.

#### 2.2.15. Statistical analysis

All cookie formulations were prepared in triplicate independent batches. Data are reported as means ± standard deviation. Statistical analysis was carried out using one-way ANOVA with Tukey's post hoc multiple comparison test *via* Statgraphics Centurion 18 software (Statgraphics Technologies Inc., USA). Significant differences between means were accepted at *p* < 0.05.

## 3. Result and discussion

### 3.1. Effects of enzymatic treatment on proximate composition of cantaloupe rind powder

The enzymatic treatment of cantaloupe rind powder resulted in significant reductions in lipid, ash, starch, and TDF contents compared to the raw counterpart (UTCP), while the protein content remained insignificantly different (Table 2). Notably, while the TDF level decreased in ETCP, the SDF level increased by 2.0-fold relative to that of UTCP. This increase is attributable to the enzymatic cleavage of glycosidic bonds within polysaccharides, yielding shorter-chain polymers or oligomers that exhibit enhanced solubility. Such modifications could influence the functional attributes of the powders, as SDFs demonstrate greater fermentability than IDFs,^6,10^ thus potentially enhancing prebiotic potential and microbial modulation in the gut. Furthermore, when fortified in food matrices, IDFs are likely to exert more pronounced steric hindrance effects compared to SDFs, thereby altering the rheological properties, texture, and stability of the final product. At the molecular level, the increase in SDF reflects the combined hydrolytic action of the two enzymes used: *endo*-1,4-β-xylanase cleaves internal β-1,4-glycosidic bonds within xylan-type hemicellulose components of the cell wall, while *endo*-1,3(4)-β-glucanase hydrolyzes internal β-1,3 and β-1,4 linkages within non-cellulosic β-glucans and β-1,4 linkages within cellulose. Since both enzymes act *endo*-lytically, hydrolysis progressively reduces the degree of polymerization of the insoluble polysaccharide network, generating shorter-chain oligosaccharides and polysaccharide fragments. These lower-molecular-weight products have a reduced capacity to sustain the extensive, cooperative intermolecular hydrogen-bonding networks that hold the polysaccharide chains in an insoluble structure, thereby conferring increased aqueous solubility.

**Table 2:** Proximate composition and functional properties of UTCP and ETCP^a^

	UTCP	ETCP
Protein (%, d.w)	4.9 ± 0.2^a^	5.1 ± 0.1^a^
Lipid (%, d.w)	5.9 ± 0.1^b^	1.6 ± 0.1^a^
Ash (%, d.w)	7.5 ± 0.4^b^	6.9 ± 0.0^a^
Carbohydrate (%, d.w)	81.7 ± 0.6^a^	86.5 ± 0.1^b^
Starch (%, d.w)	5.9 ± 0.3^b^	4.0 ± 0.2^a^
IDF (%, d.w)	34.52 ± 0.72^b^	27.67 ± 0.32^a^
SDF (%, d.w)	5.30 ± 0.17^a^	10.38 ± 0.40^b^
TDF (%, d.w)	39.82 ± 0.81^b^	38.04 ± 0.40^a^
IDF/SDF ratio	6.52 ± 0.02^b^	2.67 ± 0.12^a^
TPC (mg GAE/100 g d.w)	280.7 ± 11.5^b^	127.2 ± 0.9^a^
DPPH radical scavenging activity (µmol TE/100 g d.w)	795.6 ± 13^b^	308.0 ± 7.8^a^
Ferric reducing power (µmol TE/100 g d.w)	1188.2 ± 46.3^b^	574.9 ± 14.8^a^
WHC (g water/g d.w)	4.74 ± 0.02^a^	6.25 ± 0.08^b^
OHC (g oil/g d.w)	1.7 ± 0.1^a^	2.4 ± 0.0^b^
GAC (mg g^−1^ d.w)	140.2 ± 0.3^a^	283.9 ± 1.1^b^
CAC (mg g^−1^ d.w)	10.4 ± 0.4^a^	14.2 ± 0.2^b^

a Different letters (a–b) within the same row indicate significant differences among means (Tukey's test, *p* < 0.05). UTCP: untreated cantaloupe rind powder; ETCP: enzyme-treated cantaloupe rind powder; TPC: total phenolic content; IDF, SDF, TDF: insoluble, soluble and total dietary fiber, respectively; WHC and OHC: water and oil holding capacity, respectively; GAC and CAC: glucose and cholesterol adsorption capacity, respectively.

### 3.2. Effects of enzymatic treatment on microstructure and functional properties of cantaloupe rind powder

#### 3.2.1. Microstructural property

**Fig. 1:**
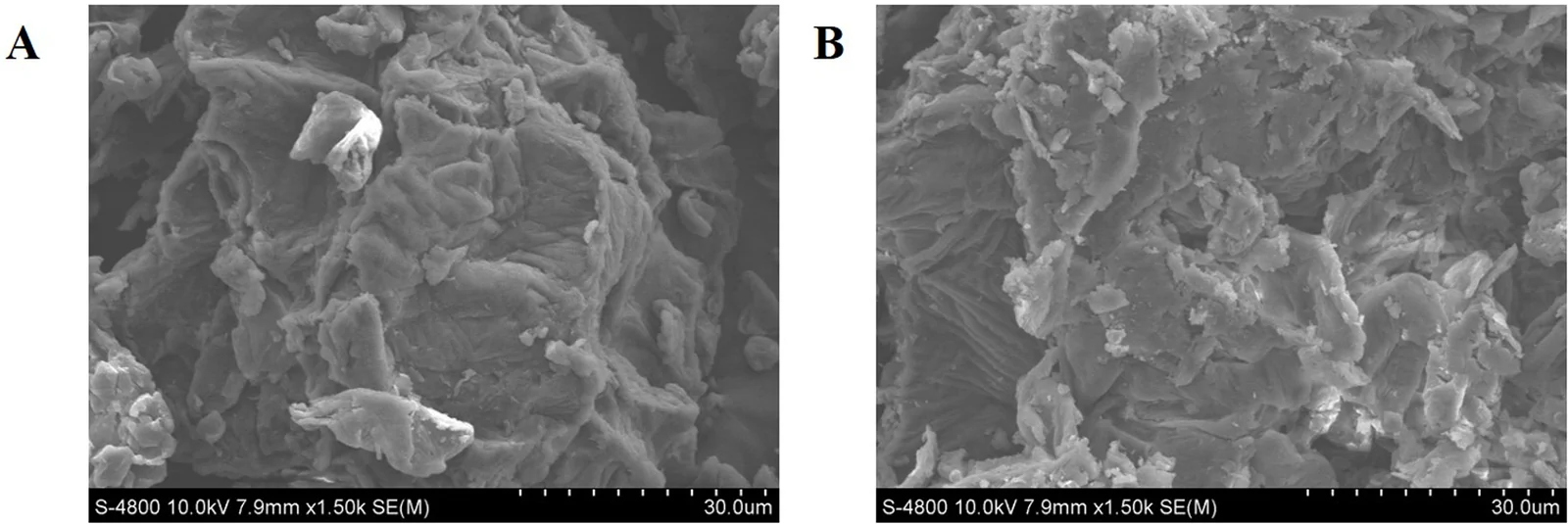
Scanning electron micrographs (SEM) of (A)- UTCP and (B)-ETCP. The magnification of the micrographs is 1500×. UTCP: untreated cantaloupe rind powder; ETCP: enzyme-treated cantaloupe rind powder.

SEM at ×1500 magnification revealed alterations in the surface morphology of UTCP following enzymatic treatment (Fig. 1). The UTCP exhibited a relatively dense and compact microstructure, with the surface appearing cohesive overall and composed of intact, continuous, sheet-like folds, reflecting the intact, tightly bound lignocellulosic network (Fig. 1A). In contrast, the ETCP displayed a modestly more irregular and fragmented surface morphology, with somewhat rougher, less continuous edges along the folded fiber layers compared to UTCP (Fig. 1B), consistent with partial structural disruption of the fiber matrix caused by enzymatic hydrolysis. These observations are consistent with enzymatic modification of plant-derived dietary fibers, as observed for soybean residue fiber^27^ and bamboo shoot fiber.^28^ Such structural modifications may increase exposed surface area, which is anticipated to enhance binding and adsorption properties by facilitating greater penetration and interaction for other molecules. Consequently, these structural modifications are expected to contribute to the physicochemical and functional properties of ETCP, including elevated WHC, OHC, GAC, and CAC.

#### 3.2.2. Antioxidant properties

The enzymatic treatment significantly reduced the total phenolic content (TPC) and antioxidant activities, as measured by the DPPH and FRAP assays, in ETCP compared to UTCP (Table 2). Specifically, the TPC decreased by approximately 2.2-fold, while the antioxidant capacity quantified *via* DPPH and FRAP assays declined by approximately 2.6-fold and 2.1-fold, respectively (Table 2). During enzyme treatment, some antioxidants could be leached into the liquid phase and could not be recovered, resulting in a reduction in TPC and antioxidant activity. Additionally, partial degradation or oxidation of phenolics under the processing conditions could further contribute to these reductions. Of these two mechanisms, leaching is considered the more likely primary contributor. The enzymatic treatment involves an extended 8 h aqueous incubation, followed by ethanol precipitation and vacuum filtration to recover the solid fraction. During this process, soluble and matrix-bound phenolics released upon hydrolysis of cellulose and hemicellulose can readily partition into the discarded liquid phase. Oxidative degradation, in contrast, would be expected to be comparatively limited under the conditions used, since the treatment was conducted at a moderate temperature (55 °C) with only brief exposure to the higher inactivation temperature (95 °C, 15 min).

#### 3.2.3. Water and oil holding capacities

The enzymatic treatment significantly enhanced both WHC and OHC of the cantaloupe rind powder (Table 2). Specifically, ETCP exhibited 1.3-fold higher WHC and 1.4-fold higher OHC compared to UTCP. The substantial increase in both WHC and OHC following the enzymatic treatment could be attributed to alterations in the dietary fiber composition and microstructure. The treatment increased the SDF content (Table 2) and disrupted the compact fiber matrix, yielding a more porous structure, providing greater exposed surface area (Fig. 1). Consequently, additional hydrophilic and lipophilic binding sites became available, improving water and oil entrapment. These improvements are consistent with studies on enzymatic hydrolysis of by-product fibers such as sweet corn milk residue,^12^ soybean residue fiber^27^ where partial degradation of IDF leads to higher WHC and OHC. A higher WHC promotes better moisture retention in food matrices, thereby reducing shrinkage caused by dehydration and increasing satiety by forming a larger bulk volume upon water adsorption.^29^ Meanwhile, enhanced OHC could be valuable in fat-rich applications, as it improves product stability while providing physiological benefits such as reduced cholesterol absorption, inhibition of lipid uptake.^30,31^ Therefore, the higher WHC and OHC of ETCP compared to UTCP indicates its greater potential as a functional ingredient in the development of fiber-enriched foods.

#### 3.2.4. Glucose and cholesterol adsorption capacities

GAC of dietary fiber correlates with its hypoglycemic efficacy by attenuating intestinal glucose absorption and modulating postprandial blood glucose levels.^32^ Similarly, CAC indicates hypolipidemic potential, which helps mitigate risks of atherosclerosis, gallstones, and cardiovascular diseases.^33^ Consistent with the improvements in WHC and OHC, the enzymatic treatment significantly enhanced both GAC and CAC of ETCP compared to UTCP (Table 2). ETCP showed 2.0-fold higher GAC and 1.4-fold higher CAC than UTCP. These significant improvements could be attributed to substantial changes in surface morphology as shown in SEM analysis, where ETCP displayed a more porous structure, facilitating physical adsorption of glucose and cholesterol molecules. Additionally, several studies report that SDF exhibits stronger glucose and cholesterol adsorption capacities than IDF.^33,34^ The results in this study align with findings on cellulase and potato residue dietary fiber modified by a mixture xylanase and cellulase, which increased SDF content and enhanced glucose and cholesterol adsorption capacities by 1.39- and 1.48-fold, respectively.^35^ Comparable effects were observed in modified fibers from bamboo shoot,^28^ and soybean residue^27^ following enzymatic or combined physico-enzymatic treatments that enhance porosity and SDF proportion. The superior glucose and cholesterol adsorption capacities of ETCP compared to UTCP demonstrate its strong potential for modulating postprandial blood glucose levels and reducing plasma and hepatic cholesterol content. This makes ETCP a promising functional ingredient for developing fiber-enriched foods with enhanced functionality.

#### 3.2.5. Growth promoting effect on *Lactobacillus acidophilus*

The growth-promoting effect of ETCP and UTCP was evaluated on *Lactobacillus acidophilus* (Table 3). The medium supplemented with 2% glucose (positive control) yielded the highest total microbial counts at both time points (7.74 log CFU mL^−1^ at 24 h and 8.37 log CFU mL^−1^ at 48 h). This is consistent with glucose acting as a readily utilizable carbon source that strongly promotes the growth of *Lactobacillus acidophilus*. In contrast, the glucose-free medium (negative control) exhibited the lowest microbial counts, with no significant change between 24 h (6.13 log CFU mL^−1^) and 48 h (6.15 log CFU mL^−1^), confirming that bacterial growth depends on the supplemented carbon source. Compared with the negative control, the media containing both UTCP and ETCP produced significantly higher microbial counts (*p* < 0.05), demonstrating their growth-promoting effect on *Lactobacillus acidophilus*. Notably, at the same supplementation level and incubation time, the medium containing enzyme-treated powder supported significantly higher probiotic counts than the medium containing untreated powder. At 48 h, the microbial growth in the UTCP-supplemented media remained statistically unchanged (*p* > 0.05) from 24 h, whereas the counts in the ETCP-supplemented media continued to increase significantly (*p* < 0.05). For ETCP-supplemented media, the microbial counts increased as the supplementation concentration increased from 2% to 4%. This concentration-dependent trend is consistent with the results reported for citrus pectin^23^ which showed stronger bacterial growth at higher supplementing concentration.

**Table 3:** Total viable count, pH, and titratable acidity (TA) of cultures supplemented with no glucose, 2% d-glucose, or 2–4% ETCP/UTCP after 24 h and 48 h of incubation^a^

		Total viable count (log CFU mL^−1^)	pH	TA (g lactic acid/100 mL)
24 h	0% d-glucose	6.13 ± 0.03^aA^	5.96 ± 0.01^hB^	0.31 ± 0.00^aA^
	2% d-glucose	7.74 ± 0.02^fA^	4.23 ± 0.01^aB^	0.77 ± 0.00^hA^
	4% ETCP	7.54 ± 0.03^eA^	4.56 ± 0.01^bB^	0.70 ± 0.01^gA^
	3% ETCP	7.45 ± 0.03^dA^	4.77 ± 0.01^cB^	0.66 ± 0.01^fA^
	2%ETCP	7.34 ± 0.03 ^cA^	4.98 ± 0.01^dB^	0.63 ± 0.01^eA^
	4% UTCP	7.15 ± 0.01^bA^	5.25 ± 0.02^eB^	0.57 ± 0.01^dA^
	3% UTCP	7.13 ± 0.01^bA^	5.33 ± 0.01^fB^	0.51 ± 0.01^cA^
	2% UTCP	7.07 ± 0.02^bA^	5.57 ± 0.02^gB^	0.46 ± 0.01^bA^
48 h	0% d-glucose	6.15 ± 0.02 ^aA^	5.71 ± 0.01^hA^	0.34 ± 0.01^aB^
	2% d-glucose	8.37 ± 0.04^fB^	3.19 ± 0.00^aA^	1.06 ± 0.02^hB^
	4% ETCP	7.92 ± 0.05^eB^	4.13 ± 0.01^bA^	0.82 ± 0.01^gB^
	3% ETCP	7.60 ± 0.04^dB^	4.46 ± 0.01^cA^	0.71 ± 0.01^fB^
	2% ETCP	7.48 ± 0.01^cB^	4.75 ± 0.00^dA^	0.66 ± 0.01^eB^
	4% UTCP	7.18 ± 0.02^bA^	5.16 ± 0.02^eA^	0.60 ± 0.00 ^dB^
	3% UTCP	7.15 ± 0.02^bA^	5.25 ± 0.02^fA^	0.54 ± 0.01^cB^
	2% UTCP	7.08 ± 0.02^bA^	5.43 ± 0.02^gA^	0.50 ± 0.02^bB^

a (a–h): Within the same time point, values with different lowercase letters indicate significant differences among cultures (*p* < 0.05). (A–B): within the same culture, values with different uppercase letters indicate significant differences between time points (*p* < 0.05). UTCP: untreated cantaloupe rind powder, ETCP: enzyme-treated cantaloupe rind powder.

The decrease in pH and increase in TA reflect the fermentative activity of probiotic bacteria, primarily through lactic acid production. The 2% glucose medium showed the lowest pH (4.23 at 24 h and 3.19 at 48 h) and highest TA (0.77% and 1.06%, respectively), consistent with its strongest microbial growth. Samples supplemented with enzyme-treated powder (especially at 4%) exhibited lower pH and higher TA than those with untreated powder at equivalent concentrations (Table 3). The changes in pH and TA between 24 h and 48 h were also more pronounced in the ETCP-supplemented cultures compared to UTCP-supplemented cultures.

Collectively, these observations indicate the greater prebiotic potential and fermentability of the enzyme-treated powder compared to the untreated powder. The enzymatic treatment enhanced the prebiotic potential of the cantaloupe rind powder by hydrolyzing complex polysaccharides into simpler, more readily fermentable oligosaccharides. This finding aligns with a previous study,^36^ in which the prebiotic effect of guava pomace was improved following enzymatic processing. Overall, these results highlight ETCP as a sustainable fiber-rich ingredient with promising prebiotic potential for functional food applications.

### 3.3. Impacts of UTCP and ETCP fortification on dough and cookie quality

#### 3.3.1. Effects on textural and rheological properties of doughs

The textural and rheological properties of control dough and dough fortified with UTCP and ETCP are summarized in Table 4. Texture profile analysis revealed a substantial increase (*p* < 0.05) in dough hardness upon addition of dietary fiber-rich powders while dough cohesiveness was significantly reduced (*p* < 0.05). The dough hardness increased by 3.55-fold and 6.44-fold in UTCP- and ETCP-fortified dough, respectively compared to the control. Meanwhile, the adhesiveness decreased markedly by 1.43-fold and 2.54-fold in UTCP- and ETCP-fortified dough, respectively compared to that of the control. The reduced adhesiveness may arise from decreased free water availability at the dough surface due to fiber hydration, thereby limiting sticky interactions.

**Table 4:** Textural and rheological properties of the doughs^a^

	Control dough	UTCP-fortified dough	ETCP-fortified dough
Hardness (N)	3.09 ± 0.07^a^	10.98 ± 0.34^b^	25.13 ± 0.38^c^
Adhesiveness (N.s)	0.33 ± 0.00^c^	0.23 ± 0.00^b^	0.13 ± 0.00^a^
*G*′ at 10 rad s^−1^ (KPa)	57.95 ± 3.69^a^	127.12 ± 8.67^b^	263.13 ± 15.58^c^
*G*″ at 10 rad s^−1^ (KPa)	27.38 ± 1.59^a^	58.84 ± 5.55^b^	118.82 ± 7.36^c^
Loss tangent (tan *δ*) at 10 rad s^−1^	0.47 ± 0.00^b^	0.46 ± 0.01 ^ab^	0.45 ± 0.00^a^

a Different letters (a–c) within the same row indicate significant differences among means (Tukey's test, *p* < 0.05). UTCP: untreated cantaloupe rind powder; ETCP: enzyme-treated cantaloupe rind powder.

**Fig. 2:**
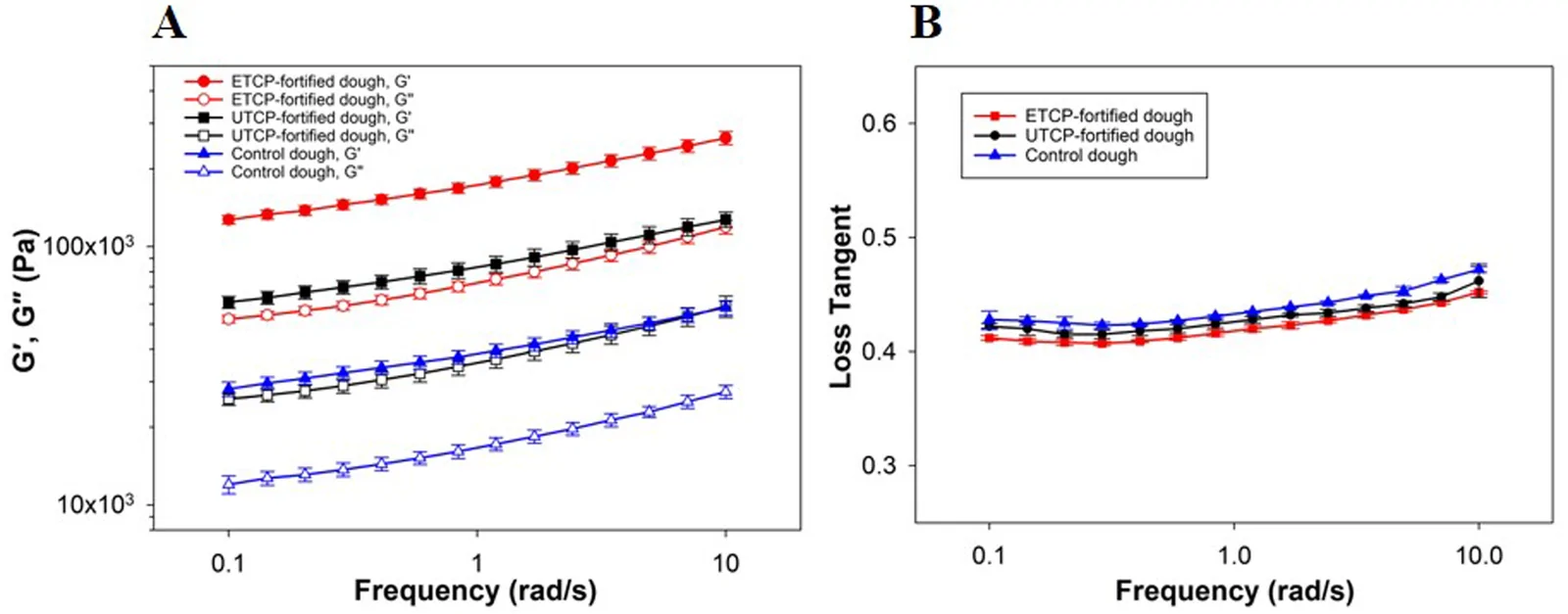
Rheological properties of cookie dough: (A) storage modulus (*G*′) and loss modulus (*G*″), and (B) loss tangent (*G*″/*G*′) for control, UTCP-fortified, and ETCP-fortified samples. Values are expressed as mean ± standard deviation (*n* = 3). UTCP: untreated cantaloupe rind powder; ETCP: enzyme-treated cantaloupe rind powder.

The storage modulus (*G*′), which reflects elastic (solid-like) behavior, and the loss modulus (*G*″), which reflects viscous (liquid-like) behavior significantly increased following UTCP and ETCP fortification (Fig. 2). The values at 10 rad s^−1^ were extracted and presented in Table 4. Compared to the control, dough fortified with ETCP showed 4.53- and 4.34-fold increases in *G*′ modulus and *G*″ modulus, respectively, while UTCP fortification resulted in a 2.19- and 2.15-fold increase. The loss tangent (tan *δ*) values of the control and UTCP-fortified dough are statistically similar, whereas this value decreased modestly in the ETCP-fortified dough (Table 4), reflecting a transition toward more elastic, solid-like properties with lower viscous dissipation in ETCP-fortified dough samples.

These modifications are consistent with the typical effects of dietary fiber fortification in wheat-based doughs.^12,37^ The incorporation of fiber-rich materials, particularly those with high water-holding capacity, competes with gluten for available water, leading to a more rigid gluten network.^38^ Insoluble fibers often act as fillers within the dough matrix, increasing resistance to deformation, resulting in higher hardness and *G*′,^37^ while soluble components may contribute to network reinforcement through hydrogen bonding or physical entanglement.^39^ The greater effects observed with ETCP compared to UTCP could be attributed its higher SDF content, greater WHC and enhanced porosity (as evidenced in SEM analysis, Fig. 1). These characteristics enabled stronger interactions with the gluten matrix, modified the secondary structures of gluten proteins,^40^ and promoted the formation of a more elastic dough structure as indicated by higher *G*′ and lower tan *δ* and a firmer dough texture. The addition of ETCP, with its higher SDF content than UTCP, produced a larger increase in *G*′ and *G*″, indicating that the additional hydrogen bonding and physical entanglement contributed by SDF, together with the higher WHC of ETCP, outweighed the loss of the insoluble filler fraction in reinforcing the dough network.

#### 3.3.2. Effects on cookie properties

##### 3.3.2.1. Chemical composition

The incorporation of UTCP and ETCP significantly reduced the starch and protein contents while slightly increasing the lipid contents in the fortified cookies compared to the control (Table 5). Notably, the TDF contents increased approximately 3.3-fold in both UTCP- and ETCP-fortified cookies compared to the control, with ETCP-fortified cookies exhibiting significantly higher SDF content than those fortified with UTCP (Table 5). Consequently, the IDF/SDF ratio was significantly lower in ETCP-fortified cookies compared to UTCP-fortified cookies. Both fortified cookie variants achieved TDF contents exceeding 6 g/100 g, qualifying for a “high in fiber” labeling under many Codex-aligned regulatory frameworks. The fortification with UTCP and ETCP significantly enhanced antioxidant capacity compared to the control, with UTCP showing a more pronounced effect. The total phenolic content (TPC) increased 3.0-fold and 2.4-fold in UTCP- and ETCP-fortified cookies, respectively. The antioxidant activity also improved markedly, with UTCP increasing DPPH radical scavenging by 3.1-fold and FRAP by 5.3-fold, compared to 2.7-fold and 2.6-fold with ETCP, respectively.

**Table 5:** Proximate composition of control, UTCP-fortified and ETCP-fortified cookies

	Control cookies	UTCP-fortified cookies	ETCP-fortified cookies
Protein (%, d.w)	7.7 ± 0.3^b^	6.0 ± 0.3^a^	6.1 ± 0.1^a^
Lipid (%, d.w)	23.8 ± 0.3^a^	24.7 ± 0.6^b^	25.0 ± 0.1^b^
Ash (%, d.w)	1.0 ± 0.0^a^	1.7 ± 0.0^c^	1.5 ± 0.0^b^
Carbohydrate (%, d.w)	67.6 ± 0.2^a^	67.7 ± 0.8^a^	67.5 ± 0.0^a^
Starch (%, d.w)	55.9 ± 1.0^c^	46.7 ± 0.4^b^	43.3 ± 0.5^a^
IDF (%, d.w)	1.4 ± 0.0^a^	6.3 ± 0.0^c^	5.2 ± 0.2^b^
SDF (%, d.w)	1.0 ± 0.0^a^	2.0 ± 0.1^b^	3.1 ± 0.1^c^
TDF (%, d.w)	2.5 ± 0.0^a^	8.33 ± 0.07^b^	8.27 ± 0.08^b^
IDF/SDF ratio	1.4 ± 0.1^a^	3.2 ± 0.3^c^	1.7 ± 0.1^b^
TPC (mg GAE/100 g d.w)	20.2 ± 1^a^	59.7 ± 2.8^c^	47.9 ± 0.9^b^
DPPH radical scavenging activity (µmol TE/100 g d.w)	131.3 ± 2.7^a^	401.7 ± 15^c^	352.7 ± 12.2^b^
Ferric reducing power (µmol TE/100 g d.w)	124.1 ± 5.2^a^	661.8 ± 23.2^c^	322.8 ± 11.0^b^

Overall, the fortification with UTCP and ETCP significantly enhanced the nutritional profile of the cookies, primarily through substantial increases in dietary fiber content and antioxidant properties compared to the control. UTCP provided a greater enhancement in antioxidant capacity, whereas ETCP was more effective at increasing SDF content.

##### 3.3.2.2. Effect on microstructure

**Fig. 3:**
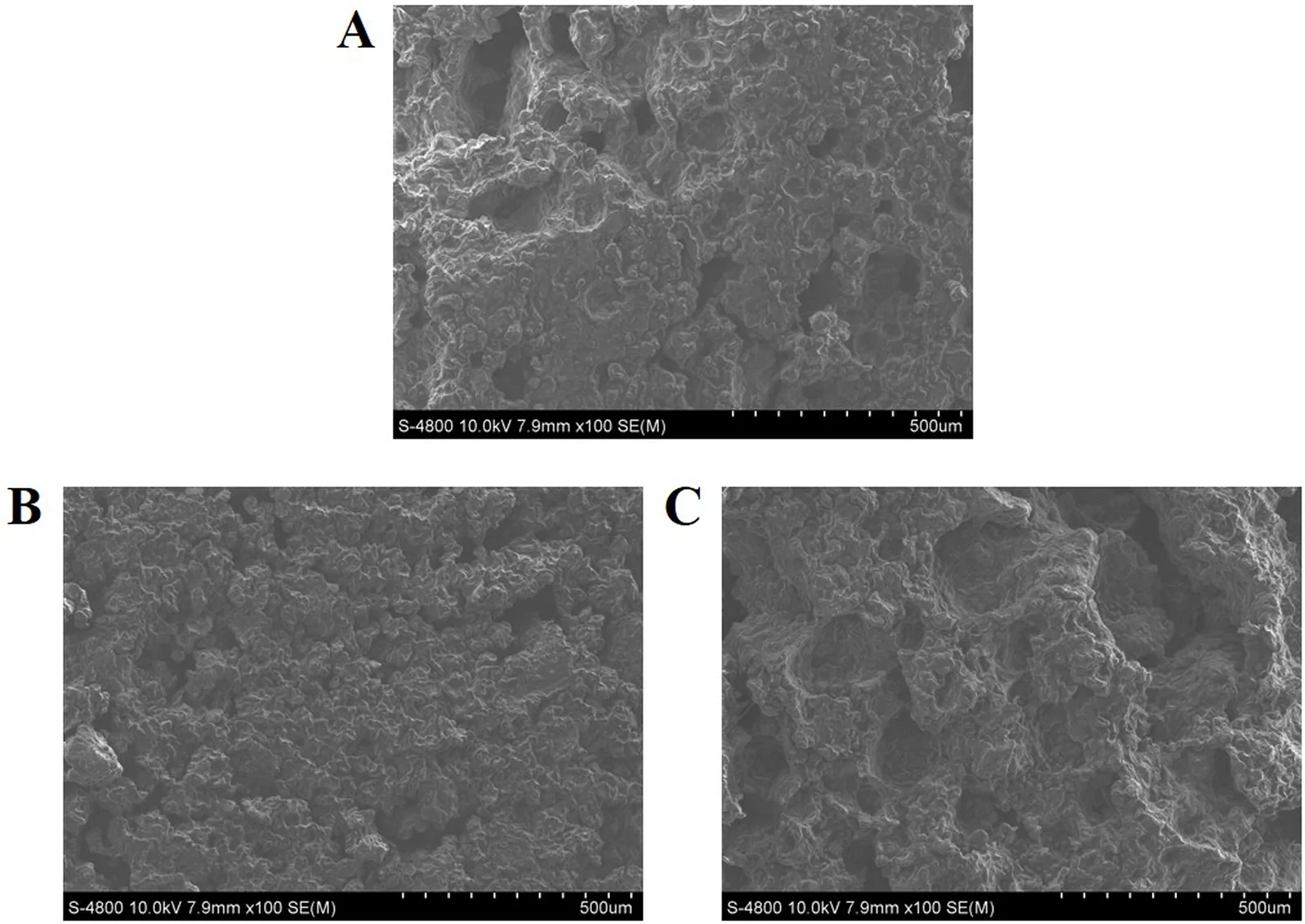
Scanning electron micrographs (SEM) of cross-section of (A) Control cookie (B) UTCP-fortified cookie and (C)- ETCP-fortified cookie. The magnification of the micrographs is 100×. UTCP: untreated cantaloupe rind powder; ETCP: enzyme-treated cantaloupe rind powder.

Cross-sectional morphological analysis of the cookies reveals distinct impact of UTCP and ETCP fortification on cookie microstructure (Fig. 3). The control cookies showed a porous structure with voids. Fortification with 20% UTCP resulted in a markedly more compact matrix with significantly smaller voids compared to the control. Starch granules in the UTCP-fortified cookies appeared less interconnected, indicating a disrupted and weakened gluten–starch network. This disruption is likely attributable to the high insoluble dietary fiber content of UTCP which physically interrupts gluten network formation during dough development. In contrast, the incorporation of 20% ETCP increased matrix porosity and generated larger voids relative to the control, suggesting enhanced gas retention during the baking process. The gluten–starch network in ETCP-fortified cookies remained relatively continuous, with starch granules well-embedded and connected. The higher soluble dietary fiber content of ETCP may promote hydrogen bonding and physical entanglement with gluten proteins and starch, increasing network viscoelasticity and gas-holding capacity, and thereby producing larger air cells and greater porosity. Overall, SEM analysis showed that UTCP fortification produced a denser cookie structure, whereas ETCP fortification resulted in a more porous structure compared to the control.

##### 3.3.2.3. Physical and textural properties

###### 3.3.2.3.1. Effect on dimensions

The incorporation of UTCP decreased both cookie diameter and thickness, while fortification with ETCP increased thickness but had no significant effect on diameter (Table 6). The thickness of the cookie is governed by the gluten structure, which is critical for retaining gas during baking.^41^ The gluten network was disrupted by the addition of UTCP with a high IDF/SDF ratio as shown by SEM analysis, which diminished gas retention and led to a decrease in thickness. Additionally, the elevated loss modulus (*G*″) in UTCP-enriched doughs relative to the control restricted dough spreading during baking, thereby decreasing cookie diameter (Table 4). On the other hand, the addition of ETCP reinforced the gluten network as indicated by SEM analysis, resulted in better gas retention and consequently higher thickness. These dimensional changes are consistent with the SEM analysis, which revealed that UTCP-fortified cookies exhibited a denser microstructure with poorly connected starch granules, whereas ETCP-fortified cookies displayed a more porous structure characterized by larger air cells and well-connected starch granules (Fig. 3). Reduction in cookie thickness is a common phenomenon observed when incorporating various dietary fiber-rich ingredients, such as untreated sweet corn milk residue,^12^ pitaya peel powder,^42^ untreated corncob powder.^43^ UTCP-added cookies displayed significant higher spread ratio (*D*/*T*) compared to the control cookies due to thickness reduction while ETCP-fortified cookies showed a comparable ratio compared to that of the control.

**Table 6:** Dimensions, textural properties, color parameters and sensory evaluation of cookies^a^

	Control cookies	UTCP-fortified cookies	ETCP-fortified cookies
**Dimensions**			
Diameter (mm)	39.58 ± 0.18^b^	38.3 ± 0.17^a^	40.19 ± 0.02^b^
Thickness (mm)	6.20 ± 0.05^b^	5.76 ± 0.03^a^	6.34 ± 0.03^c^
Spread ratio (*D*/*T*)	6.38 ± 0.07^a^	6.65 ± 0.06^b^	6.34 ± 0.03^a^

**Fracture strength**			
Hardness (N)	35.86 ± 1.3^a^	59.42 ± 0.73^c^	44.72 ± 1.79^b^
Fracturability (mm)	0.62 ± 0.04^b^	0.64 ± 0.01^b^	0.52 ± 0.01^a^

**Color parameters**			
L*	72.4 ± 0.2^c^	64.2 ± 0.2^b^	62.3 ± 0.3^a^
a*	5.1 ± 0.1^a^	6.4 ± 0.0^b^	7.5 ± 0.1^c^
b*	27.8 ± 0.0^b^	24.6 ± 0.2^a^	24.8 ± 0.2^a^
Δ*E*	0.0 ± 0.0^a^	8.9 ± 0.32^b^	10.8 ± 0.5^c^

**Overall acceptability**	7.65 ± 0.73^c^	5.40 ± 1.27^a^	6.58 ± 1.15^b^

a Different letters (a–c) within the same row indicate significant differences among means (Tukey's test, *p* < 0.05). UTCP: untreated cantaloupe rind powder; ETCP: enzyme-treated cantaloupe rind powder.

###### 3.3.2.3.2. Effect on texture

The incorporation of UTCP significantly increased cookie hardness and fracturability compared to the control (Table 6). These findings are consistent with various studies reporting increased hardness in cookies fortified with fiber-rich ingredients, such as *Cordyceps militaris* substrate,^44^ raw sweet corn milk residue powder,^12^ and dietary fiber from kinnow peels.^45^ The observed increase in hardness may be attributed to the rigid structure of insoluble dietary fibers and the high water-adsorption capacity of UTCP, which limit starch swelling, disrupt the starch–protein matrix, reduce gas retention during baking, and consequently result in a denser crumb structure (Fig. 3).

In contrast, the cookies fortified with ETCP displayed a lower hardness and fracturability compared to the UTCP-added cookies (Table 6). However, the ETCP-supplemented cookies still exhibited higher hardness but lower fracturability than the control. This behavior is likely associated with the more porous microstructures observed in ETCP-added cookies (Fig. 3), which facilitate fracture propagation.

These results suggest that the enzymatic treatment effectively mitigates the adverse textural effects associated with cantaloupe rind powder fortification. Since excessive hardness could be correlated with lower sensory scores for the texture attribute,^45^ the reduced hardness of ETCP-fortified cookies compared to UTCP-supplemented counterparts may represent an advantage in terms of improving overall sensory acceptability.

###### 3.3.2.3.3. Effects on color

The incorporation of both UTCP and ETCP decreased the lightness (L*) of the cookies (Table 6), which is consistent with previous studies reporting reduced lightness following fortification with dietary fiber–rich by-products such as bulgur brans,^46^ and kinnou peels.^45^ The red–green parameter (a*) of ETCP-incorporated cookies was higher than that of UTCP-added counterparts, and both fortified samples exhibited higher a* values compared to the control (Table 6). In contrast, the yellow–blue parameter (b*) did not differ significantly between UTCP- and ETCP-added cookies, but was lower in both samples than in the control cookies (Table 6). Overall, the total color difference (Δ*E*) was greater in ETCP-incoprporated cookies than in UTCP-supplemented counterparts, and both values exceeded 5, indicating that the color differences between the control and fortified cookies are readily perceptible to the human eye.^47^ The larger Δ*E* of the ETCP-fortified cookies relative to the UTCP-fortified counterparts represents a potential drawback, as more pronounced color deviation from a conventional cookie appearance could negatively influence sensory score for appearance/color attribute.^48^ Potential strategies to mitigate this effect in future formulations include optimizing the baking temperature or time to limit Maillard browning or incorporating a color masking ingredient (*e.g.*, cocoa cookies where a dark color is an expected sensory attribute) to reposition the product where the dark color is not perceived as a defect.

#### 3.3.3. Effect on estimated glycemic index of cookies

**Fig. 4:**
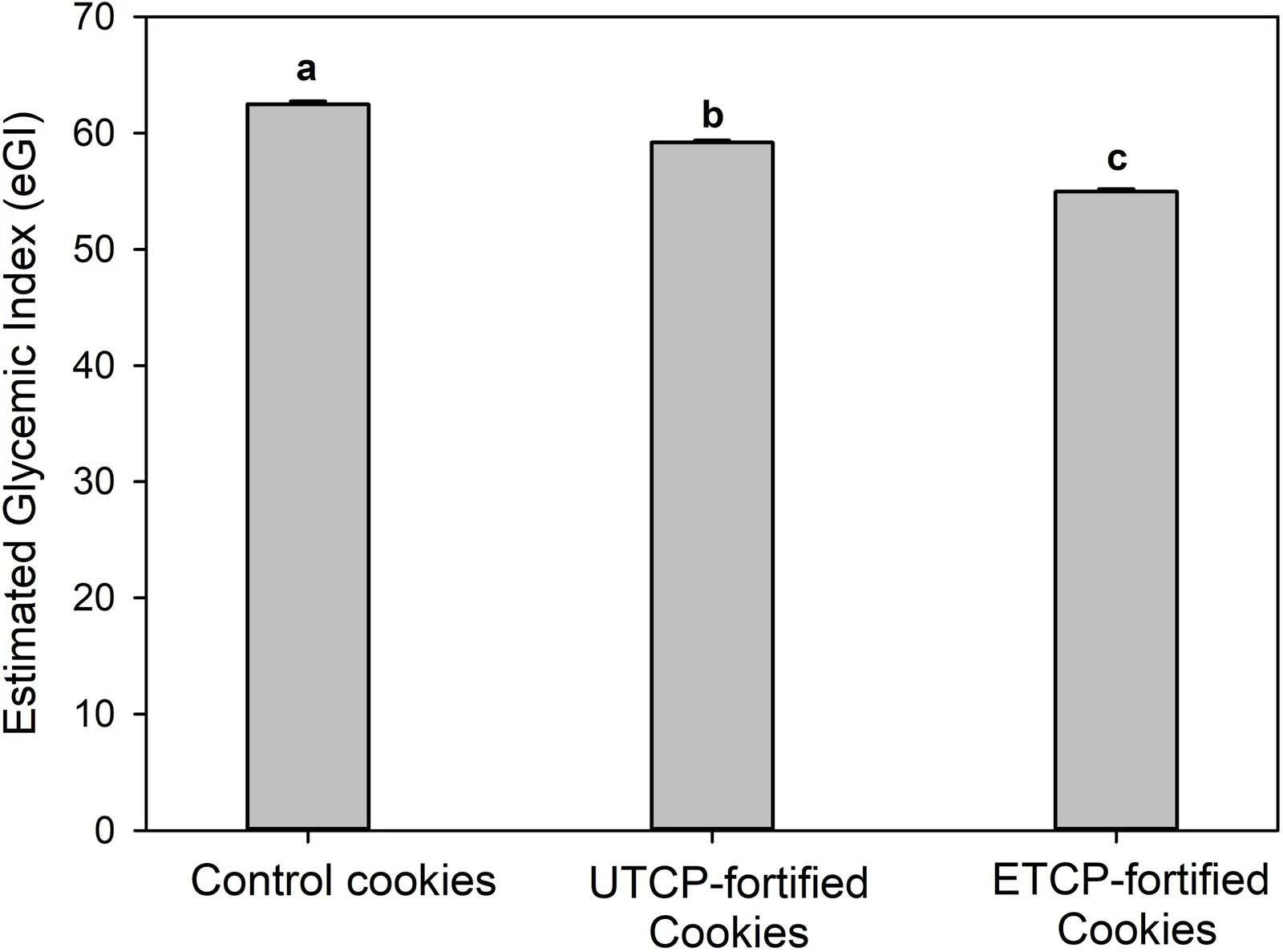
Estimated glycemic index (eGI) of control, UTCP-fortified, and ETCP-fortified cookies. Means with different superscript letters (a–c) differ significantly according to Tukey's multiple comparison test (*p* < 0.05). UTCP: untreated cantaloupe rind powder; ETCP: enzyme-treated cantaloupe rind powder.

The GI is an important nutritional indicator that quantifies the rate at which carbohydrate-containing foods increase blood glucose. Foods with a low GI produce a slower release of glucose, thereby attenuating postprandial glycemic excursions associated with obesity, type 2 diabetes, and cardiovascular disease. In this study, the fortification with UTCP and ETCP significantly lowered the eGI of cookies compared with the control (Fig. 4). The control cookies exhibited the highest eGI (62.48 ± 0.40), followed by UTCP-fortified cookies (59.21 ± 0.16), while ETCP-fortified cookies showed the lowest value (54.98 ± 0.21). This reduction is consistent with the known role of dietary fibers in slowing starch hydrolysis in carbohydrate-rich products.^49,50^ Fibers reduce α-amylase activity by limiting starch granule swellability, restricting starch gelatinization, acting as a physical entrapment that hinders enzyme access to the starch matrix, and increasing the viscosity of the digestive mixture.^49,51^ Additionally, soluble fibers can form complexes with α-amylase or starch substrates that further retard enzyme–substrate interactions, thus reducing hydrolysis rate.^51,52^ Elevated levels of soluble dietary fiber have been linked to more effective reduction of glycemic response in food systems, as shown by *in vitro* gastrointestinal simulation studies.^12,53^ While the control cookies and UTCP-fortified cookies were categorized as a medium-GI food, the cookies fortified with ETCP were classified as low-GI foods (GI < 55). Collectively, UTCP and ETCP represent effective functional ingredients for developing fiber-enriched cookies with reduced predicted glycemic impact, in which ETCP showed greater potential for regulating glycemic levels, which leads to better health results. The differential effects highlight the importance of fiber type and the potential of enzyme treatment to improve the health impact of food by-products. The higher glucose adsorption capacity of ETCP compared with UTCP, along with the significantly lower eGI observed in ETCP-fortified cookies, suggests enhanced potential for ETCP in modulating postprandial blood glucose levels. It should be emphasized that the eGI values reported here are *in vitro* predictions based on a simulated digestion model that does not account for gut microbiota activity, gastrointestinal motility, or hormonal regulation of glucose homeostasis, and should therefore be interpreted only as a preliminary screening criterion rather than a direct proxy for postprandial glycemic response, thereby supporting further evaluation of ETCP in functional foods. Therefore, future research should validate these *in vitro* findings with *in vivo* glycemic response trials.

#### 3.3.4. Effect on overall acceptability of cookies

The addition of UTCP resulted in a significant decrease of 1.37 points in the overall acceptability score of the cookies (Table 6). In contrast, incorporation of ETCP caused a milder reduction in overall acceptability, with scores 0.45 points higher than those of UTCP-fortified cookies (Table 6). This sensory improvement could be attributed to the lower hardness of ETCP-fortified cookies. Similar trends were observed in previous studies, in which enzyme-treated sweet corn milk residue powder improved sensory scores compared to the untreated counterpart, mainly due to better texture. This sensory improvement, combined with the superior functional properties of ETCP compared to UTCP, including enhanced WHC, OHC, GAC, CAC, and its ability to promote probiotic growth, highlights the strong potential of ETCP as a functional ingredient in fiber-enriched cookies.

## 4. Conclusion

Enzymatic treatment of cantaloupe rind powder using a mixture of cellulase and xylanase effectively enhanced its SDF content and porosity, resulting in improved key functional properties including WHC, OHC, GAC, CAC, and probiotic growth-promoting effect. These modifications enabled the ETCP to outperform the UTCP when incorporated into cookies at 20% substitution level. Fortification with both UTCP and ETCP significantly improved the dietary fiber content and antioxidant properties of cookies compared to the control. UTCP-fortified cookies exhibited a greater antioxidant capacity, while ETCP-cookies showed higher SDF content and a better IDF/SDF ratio. In terms of dough rheology, ETCP-fortified samples exhibited improved viscoelastic properties, evidenced by higher storage modulus (*G*′) and lower loss tangent (tan *δ*), leading to a firmer dough texture. Although both fortified cookies exhibited increased hardness relative to the control, ETCP-fortified samples exhibited lower hardness and a more porous structure than those supplemented with UTCP. Sensory evaluation indicated a reduction in overall acceptability for both fortified samples; however, ETCP caused a less pronounced decline, achieving scores 0.45 points higher than UTCP-fortified cookies. Notably, both powders lowered the estimated glycemic index (eGI) of the cookies, with ETCP demonstrating greater effectiveness. Overall, enzymatic treatment represents a simple and effective strategy to valorize cantaloupe rind, a fruit processing by-product, into a high-value functional ingredient. The superior functional properties of ETCP (enhanced WHC, OHC, GAC, CAC, and probiotic growth promotion), together with its more favorable sensory profile and stronger eGI-lowering effect compared to UTCP, highlight its strong potential for the development of fiber-enriched baked products. However, the enzymatic treatment significantly reduced the total phenolic content and antioxidant capacity of the powder, and this trade-off, together with the lower overall sensory acceptability and large total color difference (Δ*E*) observed for ETCP-fortified cookies relative to the control, indicates that enzymatic modification is not without drawbacks and that further formulation optimization is needed to improve commercial feasibility of ETCP-fortified cookies. Additionally, it should be noted that the estimated glycemic index values reported here are derived from an *in vitro* digestion model and do not capture the influence of gut microbiota, gastrointestinal motility, or hormonal regulation on the *in vivo* glycemic response. Future research should focus on optimizing the formula/preparation process to improve the sensory score of ETCP-fortified cookies, optimizing fortification levels across different bakery applications, evaluating *in vivo* hypoglycemic and hypocholesterolemia effects, and conducting comprehensive consumer sensory assessments to support the commercialization of ETCP as a sustainable functional food ingredient.

## Conflicts of interest

The authors declare no conflicts of interest.

## Data Availability

The data that support the findings of this study are available from the corresponding author upon reasonable request.
